# Results of a referral-based weight management program targeted toward children aged 2 to 6 years with obesity or severe obesity

**DOI:** 10.1186/s12887-019-1886-8

**Published:** 2019-12-19

**Authors:** Eric E. Wickel, Lamiaa Ali, Hollie Hawkins, Eden Hemming

**Affiliations:** 1 Department of Kinesiology and Rehabilitative Sciences, University of Tulsa, Tulsa, OK 74104 USA; 2 Department of Pediatrics, University of Oklahoma, Tulsa, OK 74104 USA; 3 Early Childhood Education Institute, University of Oklahoma, Tulsa, OK 74104 USA

**Keywords:** Severe obesity, Pediatric, Lifestyle, Children, Body mass index

## Abstract

**Background:**

Relatively little is known about weight management programs targeted toward young children with obesity. Using data from the Early Lifestyles Intervention program, we report outcomes from a referral-based, multi-disciplinary weight management program targeted toward children aged 2 to 6 years with obesity or severe obesity.

**Methods:**

Data from 55 children (4.5 ± 1.3 years) medically referred to the ELI program were examined in this non-randomized investigation. At baseline, a nurse collected demographic, anthropometric and clinical measures from the study child, while parents/guardians completed questionnaires regarding their child’s nutrition and activity behavior. Follow-up sessions were conducted to discuss healthy behavior strategies and collect anthropometrics from the study child. Body mass index (BMI) values were reported relative to the 95th BMI percentile (%BMI_p95_) and children were classified as obese (≥ 100% of 95th BMI percentile) or severely obese (≥ 120% of 95th BMI). Questionnaire data were analyzed to report group-level differences and to determine whether individual items predicted changes in %BMI_p95_ from baseline to follow-up. Regression models were used to examine the change in %BMI_p95_ by sex, ethnicity, and baseline body size.

**Results:**

Certain behaviors were more frequent among non-Hispanic children compared to Hispanic children (demanding certain foods), whereas other behaviors were more frequent among children with severe obesity compared to children with obesity (requesting a second helping, getting own snack and sneaking food). Greater reductions in the study child’s %BMI_p95_ were found among parents indicating their child requests a second helping, is a faster eater, or complains of being hungry. Among the combined sample, %BMI_p95_ significantly decreased from baseline to final follow-up. On average, the decrease in %BMI_p95_ did not differ by sex, ethnicity, or baseline body size.

**Conclusions:**

Modest improvements in body size were observed. Additional studies are needed to identify best practices for pediatric interventions seeking weight management.

## Background

Obesity is a global health problem associated with several physical, social, and emotional complications. Specifically, children with obesity have an increased risk of respiratory, metabolic and cardiovascular conditions [1], as well as social isolationism and depression [2]. Furthermore, longitudinal studies indicate children with obesity have an increased risk of health-related complications during adulthood [3]. Early identification of obesity, correlates of weight regulation, as well as targeted strategies to manage and prevent unnecessary weight gain should be explored, particularly in families of young children with obesity.

Based on 2015–2016 NHANES data from the United States, the percentage of children aged 2 to 5 years with obesity (≥ 95th body mass index (BMI) percentile [4]) and severe obesity (≥ 120% of the 95th BMI percentile [5, 6]) was 13.7 and 2.0%, respectively [7]. The overall prevalence of children aged 2 to 5 years with obesity increased since the 2013–2014 assessment cycle (9.3 to 13.7%; *p* = 0.011), primarily among young boys (8.5 to 14.2%; *p* = 0.018) [7]. In general, prevalence rates of obesity are consistently higher within minority subgroups (i.e., Hispanic and non-Hispanic black) [8]. Although the precise origin of obesity is unclear, interactions between an individual’s biology and their environment likely contribute toward unnecessary weight gain. Among young children, parents play a key role in the promotion of healthy behaviors, such as fruit and vegetable consumption [9], implementing rules for television viewing [10], and being physical activity [11] that likely influence weight regulation. Accordingly, family-based interventions have been endorsed for weight management [12, 13] and, to date, successful interventions have been documented. However, many intervention studies include older children and adolescents [14, 15] and consequently less is known about young children (2 to 5 years), particularly from ethnic minority subgroups who exhibit a greater range of risk factors associated with childhood obesity (e.g. higher intake of fast food, higher sugar-sweetened beverage intake, and greater likelihood of a television in the bedroom) [16].

The purpose of this study was to examine demographic and behavioral characteristics among children aged 2 to 6 years with obesity or severe obesity participating in the Early Lifestyle Intervention (ELI). A secondary purpose of this study was to describe attendance and retention characteristics among ELI participants from baseline to final follow-up.

## Methods

### Data source

Data collected from existing medical records of the ELI clinic were examined in this study. The ELI clinic is a referral-based, non-randomized weight management program targeted toward 2 to 6 year olds, and more recently expanded to also include children up to 18 years. The ELI clinic uses a coordinated, multidisciplinary approach to treat children with obesity. ELI personnel include licensed practical nurse (LPN), medical providers (a physician/ licensed nurse practitioner) who assess comorbidities, a registered dietitian who assesses diet and nutrition, and a clinical psychologist who evaluates for behavioral and psychological problems. Medical records from a total of 55 participants (44% male; aged 2 to 6 years) referred by a medical provider to the ELI clinic based on a BMI value ≥100% of the 95th percentile were examined in this analysis. Baseline and follow-up data were collected between April 2012 and March 2014. The institutional review board from the University of Oklahoma Health Sciences Center reviewed and approved the request to analyze medical records associated with the ELI clinic.

### Data collection protocol

Upon referral from a medical provider, information about the ELI clinic along with nutrition and activity questionnaires were sent to the family via mail. Parents/guardians of the study child were encouraged to complete a 20-item screener questionnaire concerning their child’s nutrition and physical activity prior to the initial ELI visit (refer to Table 2 for item description). For each statement, parents/guardians were instructed to select the response (*never, seldom, sometimes, often, or always*) that best matched their child’s typical behavior. Parents/guardians were also encouraged to complete the Family Nutrition and Physical Activity (FNPA) instrument, a 20-question screening tool that evaluates the obesogenic environment [17]. Individual questions from the FNPA were scored on a scale ranging from 1 (*never/almost never*) to 4 (*very often/always*) resulting in a maximum total score of 80 (higher scores suggest a healthier environment). Although a threshold for determining a healthy vs non-healthy environment is not available, composite scores have been used for group-level comparisons [18]. A follow-up phone call from a nurse, or patient care coordinator, was conducted to schedule an initial visit to the ELI clinic. On the first visit (from here on referred to as baseline), basic demographics (date of birth, sex, ethnicity) were reported by the parent or legal guardian and standard anthropometric (stature and body mass) and laboratory measures (blood pressure (BP) and lipid profile) were collected from the participating child. ELI personnel examined responses from the nutrition and activity questionnaires and personalized feedback was provided to each family concerning strategies to promote healthy lifestyles. Follow-up sessions were scheduled with the referred child and their parent to discuss lifestyle strategies to improve health and collect anthropometric data. The multidisciplinary team typically saw children and family members every 1 to 2 months; however, frequency of follow up was individualized based on the needs of each family.

### Measures

Stature and body mass were collected by the LPN at baseline and throughout participation in the ELI program. Stature was collected using an Ayrton stadiometer (model # S100) and recorded to the nearest 0.1 cm. Body mass was collected to the nearest 0.1 kg using a Tanita scale (model # BWB-800). Body mass index was calculated (BMI = kg/m^2^) and expressed relative to the 95th BMI percentile (%BMI_p95_). The %BMI_p95_ was used to classify participants with obesity (≥ 100% of 95th BMI) or severe obesity (≥ 120% of 95th BMI) [5, 6]. Length of participation in the ELI program (months) was determined from the baseline date to the final appointment date with ELI personnel and the change in %BMI_p95_ from baseline to final appointment date was calculated for each study child (change = final %BMI_p95_ – baseline %BMI_p95_). At baseline, the LPN also collected resting BP and blood samples. Children with systolic BP (SBP) values ≥90th percentile for sex, age, and percentile of stature were considered to have elevated SBP [19]. Blood samples were analyzed following standard techniques to report the frequency of high total cholesterol (CHOL ≥200 mg/dL), low density lipoprotein (LDL ≥130 mg/dL), and triglyceride levels (TG ≥ 100 mg/dL) [20].

### Statistical analysis

Summary characteristics (mean, standard deviation (SD), frequencies, percentages) for demographic, anthropometric, and laboratory data were derived using data from all referred children (*N* = 55). Independent *t* and χ^2^ tests were used to assess group-level differences. Items from the nutrition and physical activity screener questionnaire were examined separately to report response distributions, proportional differences by sex, ethnicity, and body size using χ^2^ tests, and to determine whether responses were related with changes in %BMI_p95_ from baseline to follow-up. The total FNPA score was calculated and used as a descriptive variable at baseline (individual item responses from the FNPA were not available to analyze).

Among the referred sample, regression analyses were conducted to predict the influence of sex, ethnicity, and baseline body size on follow up values of %BMI_p95_. Model 1 was adjusted for baseline %BMI_p95_, whereas model 2 was further adjusted for differences in the study child’s age at baseline (years) and length of ELI participation (months). Unstandardized regression coefficients (b (95% CI)) were used to interpret mean differences in follow up values of %BMI_p95_ by sex (coded 1 for girls, 0 for boys), ethnicity (coded 1 for Hispanic, 0 for non-Hispanic), and baseline body size (coded 1 for severe obesity, 0 for obese). McNemar’s test examined the consistency in body size classification from baseline to final follow-up. Statistical analyses were conducted using SPSS v24 and significance was set at *p* < 0.05.

## Results

A total of 55 children (~ 44% male) aged 2 to 6 years provided baseline measurements between April 2012 and February 2014 (Table 1). Approximately 65% of the children were Hispanic with the remaining percentage comprised of Caucasian (16%), African American (15%), and multi-ethnic/unspecified (4%) children. Overall, mean baseline values for anthropometric and laboratory measures were similar by sex and ethnicity (Hispanic vs non-Hispanic comparisons not shown in Table 1). At baseline, a majority (56%) of children were classified with obesity. The remaining 44% were classified with severe obesity (27% ≥ 120% of the 95th BMI; 17% ≥ 140% of the 95th BMI). Nearly half of the sample providing clinical measures had elevated SBP (53.7% ≥ 90th percentile) or high TG levels (54.3% ≥ 100 mg/dL) at baseline (no proportional differences were found by sex, ethnicity, or body size category). Lower proportions of the sample had high total cholesterol (8.7% ≥ 200 mg/dL) or high LDL (6.7% ≥ 130 mg/dL). Baseline %BMI_p95_ was significantly correlated with SBP (r = 0.340; *p* = 0.012) and TG (r = 0.298; *p* = 0.044). Mean FNPA total scores did not differ by sex, ethnicity or baseline body size (obese vs severely obese) (*p* > 0.05).

**Table 1:** Demographic, anthropometric and clinical characteristics of the study sample at baseline

	Combined	Male	Female	*P*-value
N (% Hispanic)	55 (65%)	24 (58%)	31 (71%)	0.328
Age, yrs	4.5 ± 1.3	4.7 ± 1.4	4.3 ± 1.3	0.256
Height, cm	107.6 ± 11.0	109.5 ± 11.1	106.2 ± 10.9	0.279
Weight, kg	26.4 ± 8.1	27.7 ± 8.7	25.4 ± 7.6	0.309
BMI, kg/m^2^	22.3 ± 3.3	22.5 ± 3.4	22.1 ± 3.2	0.600
%BMI_p95_	121.4 ± 18.2	123.6 ± 19.7	119.8 ± 17.1	0.445
% with obesity ^1^	56.4%	50.0%	61.3%	0.402
SBP, mmHg	104.9 ± 9.1	105.8 ± 9.9	104.2 ± 8.6	0.523
% ≥ 90th percentile	53.7%	60.9%	48.4%	0.363
CHOL, mg/dL	162.8 ± 31.3	165.9 ± 36.7	160.3 ± 26.4	0.556
% ≥ 200 mg/dL	8.7%	9.5%	8.0%	0.855
LDL, mg/dL	93.7 ± 30.5	96.6 ± 37.6	91.2 ± 23.1	0.562
% ≥ 130 mg/dL	6.7%	9.5%	4.2%	0.472
TG, mg/dL	122.1 ± 88.9	110.9 ± 74.1	131.4 ± 100.2	0.441
% ≥ 100 mg/dL	54.3%	42.9%	64.0%	0.152
FNPA score	56.7 ± 7.5	58.1 ± 6.7	55.2 ± 8.1	0.197

Values reported as frequencies, percentages (%) or mean ± SD

^1^Percentage of the sample classified as obese (≥ 100% of 95th BMI percentile). The remaining percentage was severely obese (≥ 120% of 95th BMI percentile)

*BMI* body mass index

*SBP* systolic blood pressure (*N* = 54, 43% male; 67% Hispanic)

*CHOL* cholesterol (*N* = 46; 46% male; 72% Hispanic)

*LDL* low density lipoprotein (*N* = 45; 47% male; 71% Hispanic)

*TG* triglyceride (N = 46, 46% male; 72% Hispanic)

*FNPA* Family Nutrition and Physical Activity (N = 45; 51% male; 64% Hispanic)

Parent/guardian responses from the nutrition and physical activity screener questionnaire are shown in Table 2. The response rate for this screener questionnaire was approximately 55%. Mean baseline %BMI_p95_ values were similar between study children with and without screener questionnaire data (*p* > 0.05). For group comparisons (e.g., by sex, ethnicity, and body size), parent/guardian responses were recoded into two categories (category 1 contained responses of *never/seldom/sometimes* and category 2 contained responses of *often/always*). Overall, response frequencies were similar by sex (*p* = 0.151 to 0.886). As shown in Table 2, certain behaviors were more frequent among non-Hispanic children compared to Hispanic children (e.g., demanding certain food or snacks, χ^2^ = 7.2; df = 1; *p* = 0.007). Other behaviors were more frequent among children with severe obesity compared to children with obesity at baseline (e.g., child requesting a second helping (χ^2^ = 5.0; df = 1; *p* = 0.025), getting their own snack (χ^2^ = 7.7; df = 1; *p* = 0.005), and sneaking or hiding food (χ^2^ = 5.3; df = 1; *p* = 0.022)). Although we did find some baseline behaviors associated with a greater reduction in %BMI_p95_ from baseline to follow-up (e.g., parents/guardians *often/always* reporting their child requests a second helping (Mann-Whitney U = 43; *p* = 0.019), are faster eaters (Mann-Whitney U = 65; *p* = 0.050), or they complain of being hungry (Mann-Whitney U = 8; *p* = 0.001)) some caution is warranted given the responses were from a subset of the entire study sample.

**Table 2:** Response distribution for 20-item nutrition and activity behavior questionnaire

	N	Percent (%) reporting	Group level differences in *Often* or *Always* response at baseline
		*Often* or *Always*	
Drinks juice between meals.	30	20.0	–
Eats junk food.	30	23.3	–
Eats more than 2 snacks between meals a day.	30	33.3	–
Requests second helping.	30	30.0	Severely obese > obese(χ^2^ = 5.0; df = 1; *p* = 0.025)
Is a fast eater.	30	50.0	–
Regularly eats fast-food.	30	10.0	–
Gets own snacks.	29	44.8	Severely obese > obese(χ^2^ = 7.7; df = 1; p = 0.005)
Sneaks or hides food.	30	13.3	Severely obese > obese (χ^2^ = 5.3; df = 1; p = 0.022)
Demands certain food or snacks.	28	32.1	Non-Hispanic > Hispanic (χ^2^ = 7.2; df = 1; p = 0.007)
Refuses to eat fruit or vegetables.	30	20.0	–
Refuses to eat dairy products.	30	3.3	–
Eats in front of the television.	30	16.7	–
Complains of being hungry.	25	24.0	–
Becomes angry when demands for food are not met.	25	20.0	–
Wants to ride in the stroller.	25	4.0	–
Watches more than 2 h of TV each day.	30	26.7	–
Does not want to go outside to play.	30	13.3	–
Prefers quiet activities.	30	16.7	–
Has a TV in the bedroom.	25	36.0	–
Parents and family members disagree on what, when, where, and how much the child should eat.	24	4.2	–

Values represent number or percentage (%)

On average, ELI participants attended 6.4 visits (SD = 3.8) across 12.5 months (SD = 6.4) with no mean differences by sex, ethnicity or baseline body size (*p* > 0.05) (Table 3). Among this sample, four children participated less than 3 months, whereas three children participated in ELI more than 22 months.

**Table 3:** Characteristics of ELI participation for the entire sample and by sex, ethnicity, and baseline body size

		Visits post-baseline		Length of follow-up, months		Frequency distribution for final follow-up length, months			
	N	mean ± SD	(min, max)	mean ± SD	(min, max)	0.5–5.9	6.0–11.9	12.0–17.9	18+
All	55	6.4 ± 3.8	[1, 16]	12.5 ± 6.4	(0.6, 23.2)	12	13	18	12
Sex									
Boys	24	6.0 ± 3.8	[1, 16]	12.8 ± 6.8	(1.8, 22.7)	6	5	7	6
Girls	31	6.6 ± 3.7	[1, 14]	12.3 ± 6.3	(0.6, 23.2)	6	8	11	6
Ethnicity									
Non-Hispanic	19	5.4 ± 3.2	[2, 14]	11.0 ± 6.5	(0.6, 23.2)	6	4	7	2
Hispanic	36	6.8 ± 4.0	[1, 16]	13.3 ± 6.4	(1.2, 22.7)	6	9	11	10
Baseline body size									
Obese	31	6.2 ± 3.9	[1, 14]	12.4 ± 6.8	(0.6, 23.2)	9	5	9	8
Severely obese	24	6.5 ± 3.6	[1, 16]	12.6 ± 6.1	(1.2, 22.7)	3	8	9	4

Among the combined sample, %BMI_p95_ decreased from 121.4 (SD = 18.2) at baseline to 118.6 (SD = 16.6) at follow-up (mean decrease = 2.9, 95% confidence interval (CI) = 0.5 to 5.2, *p* = 0.017). As shown in Table 4, mean change scores and regression coefficients revealed similarities by sex and ethnicity. Baseline %BMI_p95_ was inversely related with the change in %BMI_p95_ (r = − 0.416; *p* = 0.002) suggesting participants with higher baseline %BMI_p95_ values experienced greater reductions in %BMI_p95_ than those with lower baseline %BMI_p95_ values. Although we found a significant mean difference in %BMI_p95_ change scores between children with obesity (mean change = − 0.6) and severe obesity (mean change = − 5.8) (mean difference = 5.2 (0.2, 10.2)), the finding was likely attributed to regression to the mean [21].

**Table 4:** Descriptive and group level changes in %BMI_p95_ by sex, ethnicity and baseline body size

		%BMI_p95_ scores			Estimated regression coefficient (b)	
	N	Baseline	Follow up	Change score	Model 1	Model 2
Sex						
Boys	24	123.6 ± 19.7	121.2 ± 18.8	−2.4 ± 7.4		
Girls	31	119.8 ± 17.1	116.5 ± 14.7	−3.3 ± 9.7		
Mean difference		3.8 (−6.2, 13.8)	4.7 (−4.3, 13.8)	0.9 (− 3.9, 5.7)	−1.7 (−6.1, 2.7)	−2.0 (− 6.5, 2.4)
P-value		0.445	0.302	0.712	0.452	0.365
Ethnicity						
Non-Hispanic	19	124.6 ± 19.3	121.7 ± 15.9	−2.9 ± 8.7		
Hispanic	36	119.8 ± 17.7	116.9 ± 17.0	−2.9 ± 8.8		
Mean difference		4.8 (−5.5, 15.2)	4.9 (−4.5, 14.3)	0.0 (−5.0, 5.0)	−1.0 (−5.6, 3.6)	−0.7 (− 5.4, 4.1)
*P*-value		0.353	0.306	0.994	0.667	0.780
Baseline body size						
Obese	31	108.7 ± 5.4	108.0 ± 8.4	−0.6 ± 5.5		
Severely obese	24	138.0 ± 15.4	132.2 ± 14.6	−5.8 ± 11.0		
Mean difference		29.3 (3.3, 22.5)	24.1 (3.3, 17.3)	5.2 (0.2, 10.2)	1.8 (−5.6, 9.2)	2.1 (−5.3, 9.6)
P-value		< 0.001	< 0.001	0.044	0.629	0.564

Values are mean ± SD or mean (95% CI)

Estimated regression coefficient for sex, ethnicity and baseline body size was adjusted for baseline %BMI_p95_ in Model 1 and further adjusted for the study child’s age at baseline (years) and length of ELI participation (months) in Model 2

Overall, seven children (nearly 13% of the study sample) decreased below 100% of the age- and sex-specific 95th BMI percentile (i.e., transitioned from obese to overweight). Among the remaining 48 children, McNemar’s test was not statistically significant (*p* > 0.05) indicating the proportion of children with severe obesity was similar at baseline and final follow-up.

## Discussion

This study examined the change in %BMI_p95_ among 2 to 6 year olds participating in a referral-based weight management program. On average, ELI participants attended nearly six visits over an approximate 1-year period. Among the combined sample, %BMI_p95_ significantly decreased from baseline to final follow-up with no group-level differences by sex, ethnicity or baseline body size. Although the study design lacked a control group to evaluate the effectiveness of the ELI program, the findings contribute to a burgeoning area of study regarding the identification, treatment, and participation of young children with obesity and severe obesity in referral-based weight management programs.

Immediate [1] and long-term [3] health complications exist in children with excess adiposity; as such, considerable attention has been placed on early detection and treatment. Currently, a variety of body size metrics have been used in clinical and non-clinical settings to assess body size, examine longitudinal trajectories and examine the effectiveness of weight management programs using randomized [22–27] and non-randomized designs [15, 28, 29]. The Centers for Disease Control and Prevention (CDC) BMI-for-age growth charts [30, 31] are commonly used to identify children as overweight (85th ≤ BMI percentile <95th) or obese (BMI percentile ≥95th) based on percentile ranking [32]. For a given BMI percentile, a BMI *z* score can be estimated to report the number of standard deviation units above or below a reference median value [33]. A *z* score of zero equals the 50th percentile, whereas *z* scores of + 2 and + 3 are used to identify children as overweight or obese, respectively [34]. Although BMI percentiles and *z* scores are useful among normal weight children and children with moderate obesity, their use among children with severe obesity may be misleading and prone to erroneous conclusions due to the compression of high BMI values into a narrow range of percentiles (e.g., 95th to 100th) and *z* scores [5, 35, 36].

Analyzing data used to construct the BMI-for-age growth charts, Flegal and colleagues [5] found *z* scores were inaccurate above the maximum reference 97th BMI percentile. Freedman and colleagues [35] observed similar limitations of *z* scores among young children (ages 2 to 4 years) with severe obesity. In their analysis of nearly 8.7 million children, *z* scores varied by more than 3-fold among children of similar body size. Furthermore, in an analysis involving NHANES data from 1999 to 2014, weak associations involving BMI *z* scores with waist circumference (r = 0.10) and triceps skinfold thickness (r = 0.07) were observed among children with severe obesity [36]. In contrast, relatively stronger associations were found involving %BMI_p95_ with waist circumference (r = 0.55) and triceps skinfold thickness (r = 0.32) [36]. Although a consensus body size metric isn’t available, %BMI_p95_ offers a flexible option to assess body size across the entire range of the BMI spectrum [37].

Among this sample of young children, we observed a modest reduction in %BMI_p95_ (nearly 2.4% reduction from baseline to follow up). Among adults, a 3–5% body weight reduction is clinically meaningful [38]; however, in children this information is unknown, perhaps due to varying BMI metrics across studies. Despite the relatively small change in %BMI_p95_ and a high proportion of children persisting in the same body size category at baseline and final follow-up (21 out of 25 children remained in obese category; 20 out of 23 children remained in the severe obese category), it is possible that other health-related benefits may have occurred. For example, Kalarchian et al. [14] reported a greater reduction in percent body fat and total fat mass among young children randomly allocated to an intervention group (compared to a usual care group) in the absence of changes to BMI or percentage of overweight. Similarly, Taveras et al. [26] noted a greater reduction in screen time (but not BMI) among young children assigned to an intervention group compared to peers in a usual care group. Owing to the design of the current study, we were unable to assess changes to health-related indicators (i.e., SBP and blood lipids) and behaviors (i.e., increased physical activity or reduced screen time). Future randomized controlled trials conducted in the ELI clinic will address this limitation.

Although we were unable to report program effectiveness, the current study provides useful information about attendance and retention characteristics among families participating in a referral-based weight management clinic. On average, participants attended nearly six visits over 12.5 months with no observed differences by sex, ethnicity or baseline body size. This finding suggests the ELI program was acceptable for many families, but highlights the need for systematic studies exploring determinants of program attendance and retention. In a prior pediatric weight management study, Skelton et al. [39] conducted semi-structured interviews with parents and children to assess program satisfaction and attrition. Overall, parents and children reported positive experiences with the program and children reportedly enjoyed talking to someone about their weight. Among their sample, program attrition was primarily attributable to logistical issues, such as time, transportation and clinic hours. Similar barriers likely exist across other participant pools; therefore, weight management clinics are encouraged to consider creative opportunities to overcome these challenges to maximize program participation.

Strengths of this study included the multidisciplinary team approach, the use of %BMI_p95_ to report changes in body size, and the average length of ELI participation (12.5 months). Limitations also existed in this study. Specifically, this was a non-randomized, observational analysis; therefore, in the absence of a control group, we were unable to test the overall effectiveness of the ELI program. Future studies are encouraged to employ a randomized design to assess program effectiveness and include a larger sample size to allow for associations involving baseline clinical characteristics with changes in BMI. Although our sample was ethnically diverse, the relatively low sample size prohibited us from exploring the influence of ethnicity on the change in %BMI_p95_ beyond a two-group approach (i.e., Hispanic vs non-Hispanic). A recent study [40] reported some effectiveness of a 10-week family-based, culturally tailored randomized intervention to reduce BMI and BMI z-scores among Latino children, but additional studies that recognize cultural differences in obesity-related risk factors are needed given that ethnic minorities (African American, Hispanic/Latino, and Asian) have been shown to report lower satisfaction, compared to Caucasians, regarding 1) the amount of advice on nutrition and physical activity, 2) the time spent discussing physical activity, and 3) the overall quality of physical activity advice [41].

## Conclusions

In summary, we found a modest reduction in %BMI_p95_ among our study sample. Future studies of young children (2 to 6 years old) using randomized designs are needed to identify best practices for intervention type (family-based vs parent-based) and frequency. To improve intervention participation, additional studies are necessary to examine social and financial barriers, as well as time and transportation constraints.

## Data Availability

The datasets used and/or analyzed during the current study are available from the corresponding author on reasonable request.

## Abbreviations

BMI: body mass index
BP: blood pressure
CDC: Centers for Disease Control and Prevention
CHOL: cholesterol
CI: confidence interval
df: degrees of freedom
ELI: Early Lifestyles Intervention
FNPA: Family Nutrition and Physical Activity
LDL: low-density lipoprotein
LPN: licensed practical nurse
NHANES: National Health and Nutrition Examination Survey
SBP: systolic blood pressure
SD: standard deviation
TG: triglyceride
